# Involvement in the criminal justice system among attendees of an urban mental health center

**DOI:** 10.1186/s40352-015-0017-3

**Published:** 2015-02-25

**Authors:** Allyson Anderson, Silke von Esenwein, Anne Spaulding, Benjamin Druss

**Affiliations:** 1 School of Public Health, Health Policy and Management Department, Emory University, 1518 Clifton Rd NE, Atlanta, GA 30322 USA; 2 School of Public Health, Epidemiology Department, Emory University, 1518 Clifton Rd NE, Atlanta, GA 30322 USA

**Keywords:** SMI, Incarceration, Urban, Education, Race, Risk Factors

## Abstract

**Background:**

Incarceration rates for people with serious mental illnesses are higher than the general population. However, research has been limited in regards to patterns of incarcerations for patients treated in public mental health settings. This study examines differences in lifetime imprisonment rates among patients of a U.S. urban Community Mental Health Center (CMHC) and national samples, within gender, race and education subgroups.

**Findings:**

Participants were interviewed about their criminal history. Analyses compared lifetime incarceration history in this sample to a group with similar demographics. A majority (69.6%) of the sample had been incarcerated and 34.0% had been incarcerated with a felony charge as compared with 2.7% expected for the control sample.

**Conclusion:**

Within every racial and educational subgroup, incarceration rates were high compared to the general population. Though racial and educational factors partly explained added incarceration risk, presence of a serious mental disorder heightened the incarceration risk within all strata in this public sector setting.

Every year, approximately 1 million arrests in the United States involve a person with a Serious Mental Illness (SMI) (Fisher et al., 2011). Some literature has shown that sociodemographic factors (i.e., substance abuse history, lack of employment, or homelessness) at least partly explain elevated arrest rates among this population (Fisher et al., 2011; Greenberg et al., 2011; White et al., 2006; Draine et al., 2002). While research has examined the interaction of incarceration risk factors in the general population, there is no clear understanding how risk factors and SMI may influence criminal justice system (CJS) involvement in this population. An SMI diagnosis may increase the relative incarceration risk and CJS involvement for persons that are already facing a number of socio-economic challenges. For instance, in a study examining the National Longitudinal Surveys, Western and Pettit (2009) calculated cumulative risk of imprisonment from a national probability sample using longitudinal data and life-table methods that were displayed in terms of sex, race, and education. The goal of the study was to understand the degree to which having an SMI in a public sector CMHC is a risk factor for incarceration over and above education, sex and race in comparison to the general population.

Western and Pettit concluded that there was a concentration of incarceration among low education and low-skilled black men. Though, the research addressed education, sex, and race as risk factors for incarceration, it did not examine SMI as an additional factor placing individual in the CJS and its potential influence on incarceration rates.

## Methods

Patients participating in a trial of medical care management were recruited from an urban CMHC serving the poor and uninsured in metro Atlanta (Druss et al., 2010). Inclusion criteria for the project required that an individual had a SMI diagnosis and the capacity to consent. The current analysis used baseline data from the larger study. The participants were asked about their involvement in the CJS. They were asked to report any incarceration history and past offenses. Participants were not asked to distinguish between jail detention and prison. In order to approximate rates of incarceration in a prison (i.e., imprisonment), we created a new variable, “likely in prison”. Persons who self-reported past incarceration as an adult and a felony charge were grouped into this variable. “Likely in prison” could thus be comparable to the variable used in Western and Pettit (2009) of “imprisonment”.

The data was analyzed using the statistical software PASW Statistics 18 (formerly SPSS) (IBM SPSS, Inc., 2009). Observed and expected values by gender, race, and educational attainment were compared to national imprisonment statistics taken from Western and Pettit (2009), which they calculated using data from the National Longitudinal Survey of Youth (NLSY) 79. These values analyzed imprisonment by birth cohort. The NLSY 89 was chosen because this cohort, persons born 1955-1959, contained the average birth year of participants. Western and Pettit (2009) used life-table methods to generate cumulative risk of imprisonment based on race, gender, and educational attainment in the presence of mortality. Their analysis provided a national rate comparison and expected values were calculated for each group from the sample data. Chi-square tests were performed to test the significance of the difference between the observed and the expected incarceration rates provided by Western and Pettit (2009) using OpenEpi (Dean et al., 2006).

## Findings

The sample consisted of 191 participants (see Table 1). Males represented 48.6% (n = 93) of the sample. Participants were 46.94 years of age on average. One-hundred and forty-nine (78.0%) participants self-identified as black and 33 (17.3%) self-identified as white. The majority (n = 128, 67.0%) of participants had at least a high school education. Primary psychiatric diagnoses were depression (n = 169, 88.0%) and schizophrenia/schizoaffective disorder (n = 80, 41.9%). In addition, most participants (n = 171, 89.5%) had an annual income of less than $10,000; and 50 (26.2%) of those individuals had no income at all.

**Table 1:** **Community mental health center sample descriptive statistics**

			**Never Incarcerated**		**Ever Incarcerated**		**Likely ever in prison**	
	**Total**	**%**	**N**	**%**	**N**	**%**	**N**	**%**
**Total**	191		58	30.4	133	69.6	65	34.0
**Gender**								
Male	93	48.6	15	16.1	78	83.9	43	46.2
Female	98	51.3	43	43.9	55	56.1	22	22.4
**Age**								
Minimum	24		24		24		28	
Maximum	74		74		64		60	
Mean	46.94		48.66		46.28		46.54	
**Age Group**								
20-29	5	2.6	1	20.0	4	80.0	1	20.0
30-39	19	9.9	6	31.6	13	68.4	7	36.8
40-49	98	51.3	25	25.5	73	74.5	33	33.7
50-59	57	29.8	20	35.1	37	64.9	22	38.6
60-69	11	5.8	5	45.5	6	54.5	2	18.2
70+	1	0.5	1	100.0	0	0.0	0	0.0
**Race**								
White	33	17.3	9	27.3	24	72.7	11	33.3
Black	149	78.0	47	31.5	102	68.5	51	34.2
Other	9	4.7	2	22.2	7	77.8	3	33.3
**Education Level**								
Less than high school	60	31.4	15	25.0	45	75.0	26	43.3
High school diploma/GED	82	42.9	27	32.9	55	67.1	28	34.1
Some college or greater	46	24.1	15	32.6	31	67.4	10	21.7
Unknown	3	1.6	1	33.3	2	66.7	1	33.3
**Employment**								
Disability	73	38.2	31	42.5	42	57.5	18	24.7
Unemployed	94	49.2	18	19.1	76	80.9	42	44.7
Other	24	12.6	15	62.5	9	37.5	5	20.8
**Annual Income**								
No Income	50	26.2	10	20.0	40	80.0	25	50.0
1-5000	58	30.4	15	25.9	43	74.1	18	31.0
5001-10000	63	33.0	28	44.4	35	55.6	18	28.6
10001-15000	12	6.3	2	16.7	10	83.3	3	25.0
>15000	5	2.6	2	40.0	3	60.0	1	20.0
**Mental Health**								
Schizophrenia/schizoaffective disorder	80	41.9	20	25.0	60	75.0	30	37.5
PTSD	52	27.2	15	28.8	37	71.2	19	36.5
Depression	168	88.0	54	32.1	114	67.9	55	32.7
Bipolar/Manic depression	51	26.7	11	21.6	40	78.4	14	27.5
Anxiety disorder	80	41.9	21	26.3	59	73.8	28	35.0
Alcohol abuse	71	37.2	6	8.5	65	91.5	34	47.9
Drug Abuse	77	40.3	8	10.4	69	89.6	34	44.2
Other Emotional conditions	9	4.7	4	44.4	5	55.6	2	22.2

Description of the sample population self-reported by participants from a CMHC in Atlanta, GA.

A total of 156 (81.7%) participants reported prior arrests, 133 (69.6%) had been incarcerated in their lifetime, and 18 (9.4%) were currently involved in legal proceedings. Participants self-reported felony offenses including violent crimes (i.e. murder, aggravated assault, and weapons offense) (n = 24, 10.7%), property crimes (n = 22, 9.8%), drug charges (n = 13, 5.8%), obstruction (n = 4, 1.8%), and DUI felony (n = 2, 0.9%). Misdemeanor charges included property crimes (n = 36, 16.0%), crimes against public order (n = 23, 10.22%), DUI (n = 20, 8.89%), violent crimes (i.e. assault and battery) (n = 14, 6.22%), probation violation (n = 3, 1.33%) and sex crimes (i.e. prostitution and indecent exposure) (n = 2, 0.89%). A total of 65 (34.0%) participants reported past incarceration as an adult and a felony charge—these were the participants who were “likely in prison”.

Rates of “likely in prison” for each demographic group from the sample population were greater than the published expected lifetime risk of imprisonment (see Table 2). According to the NLSY89 statistic calculated by Western and Pettit (2009), twelve participants from the sample were expected to have a history of imprisonment. The sample displayed a significantly higher rate of imprisonment in comparison to the general population statistics (*χ* ^2^ = 41.9, df = 1, p < 0.01). Nearly 49% of black men in this sample were likely in prison in their lifetime, a risk ratio three times the national average of imprisonment for black men (14.09%, p < 0.01); and 22.2% of black women were likely in prison in their lifetime, which is 12 times the national average of imprisonment for black women (1.79%, p < 0.01). White men and women were not expected to have any prior imprisonments in this sample. However, 40% (p < 0.01) of white men were likely imprisoned in comparison to the 2.3% lifetime imprisonment risk for white men; and 23.1% (p < 0.01) of white women in comparison to the 0.25% lifetime imprisonment risk.

**Table 2:** Observed Incarceration and Calculated Imprisonment versus Expected Lifetime Imprisonment Risk for CMHC Sample Population in Atlanta GA

		**Total in CMHC**	**Incarceration in CMHC population**		**Likely Ever in Prison**		**NLSY89 Cumulative Risk of Imprisonment**	**Expected in CMHC**	
			**N**	**%**	**N**	**%**	**%**	**N**	**P-value**
Black men	Less than High school	26	24	92.3	15	57.8	28.34	7	
	High school or equivalent	23	18	78.3	9	43.5	12.64	3	
	Some college or higher	19	17	89.5	9	47.4	4.97	1	
	All black men	68	59	86.8	33	48.5	14.09^*^	10	<0.01
White men	Less than High school	7	5	71.4	3	42.9	8.57	1	
	High school or equivalent	9	8	88.9	5	55.6	2.50	0	
	Some college or higher	4	3	75	0	0	0.68	0	
	All white men	20	16	80	8	40	2.30^*^	1	<0.01
Black women	Less than High school	24	14	58.3	7	29.0	4.93	1	
	High school or equivalent	40	20	50	10	25.0	1.36	1	
	Some college or higher	17	9	52.9	1	5.9	0.83	0	
	All black women	81	43	53.1	18	22.2	1.79^*^	2	<0.01
White women	Less than High school	2	1	50	0	0	0.81	0	
	High school or equivalent	5	5	100	3	60.0	0.30	0	
	Some college or higher	6	2	33.3	0	0	0.09	0	
	All white women	13	8	61.5	3	23.1	0.25^*^	0	<0.01

*These values for cumulative risk of imprisonment represent people 18-64 years of age.

*Note:* NLSY 1989 cohort is born 1955-1959. NLSY values were calculated by Western B & Pettit B (2009). When comparing “Likely ever in prison” of the sample population to the NLSY expected imprisonment in this population, the p-value was <0.01. When analyzing imprisonment for each racial, gender group, the p-value was <0.01 for each group.

In analyzing education in this population, formerly incarcerated individuals with a high school education (n = 51, 66.2%) and those with some college or higher (n = 31, 67.4%) had comparable incarceration rates. However, individuals with less than a high school education (n = 44, 91.5%) had significantly greater incarceration rates. The negative correlation between education and imprisonment indicated that individuals with more education were less likely to have an imprisonment history. Though, it was observed that individuals with SMI have a significantly greater risk of incarceration history than the general population; education may be a protective factor from imprisonment in this population.

## Discussion

The rates of ever being incarcerated and/or likely imprisoned in this sample were significantly higher than the general population by race, gender, and educational level, by a factor of 5. As expected, men had higher incarceration rates than women. Comparable to Western and Pettit (2009), men and those with less than a high school education have a greater risk of imprisonment than others within the sample population. Unlike other literature, race did not significantly affect incarceration risk since incarceration history was comparable between races, which may be attributable to the overrepresentation of African-Americans in the sample.

Incarceration rates were significantly higher in each demographic category. Thus, SMI may play a mediating role in incarceration within this population over and above race, education, and gender, possibly by placing them in these at-risk groups of being impoverished and less educated. In comparison to Western and Pettit’s (2009) general population, incarceration is a greater risk for individuals with an SMI as they encounter CJS more than the general population. Historically, this population has had significantly lower educational attainment than individuals without mental disorders (Breslau et al., 2008) and about 90% of individuals in this sample lived below the 2005 U.S. poverty line (US Census Bureau, 2011). From Western and Pettit’s conclusions, individuals with low education and low wages have a concentrated rate of incarceration. Though they may still experience incarceration or arrests at approximately the same rate across education statuses, educational attainment may protect this population from imprisonment. The combination of these risk factors in this population show that these individuals are not only poor and less educated, but they also have to manage a mental illness that may contribute to their encounters with the CJS.

Several limitations should be noted. First, the sample of patients was from only one public sector outpatient clinic, in a state with a high incarceration and poverty rates, limiting the generalizability of the findings (Pew Center on the States, 2012). Second, the dependent variable of incarceration was self-reported; respondents possibly misreported or underreported their involvement in the CJS. A large literature surveyed in Spelman (1994) examines the validity of self-reports of imprisonment in which infrequent offenders underreport criminal activity, frequent offenders exaggerate criminal involvement, and offenders are less likely to disclose crimes for serious offenses. However, participants’ reports of past serious offenses were useful to analyze how this population is affected by criminal involvement and an issue that needs to be further addressed and understood. Lastly, Western and Pettit (2009) calculated these values for first incarceration as an age-specific cumulative risk of incarceration by age 35 and the average age of the study sample is 46.94 years. These limitations notwithstanding, incarceration history in this sample is significantly higher than in the general population lifetime risk of incarceration, even when compared to other high-risk sociodemographic strata. Promoting education and skills training in this population could potentially alter the outcomes of CJS involvement. In addition, it is important to further investigate the mediating roles of poverty and mental diagnosis over and above these demographic risks for incarceration. This study shows that having an SMI increases the likelihood of imprisonment for individuals regardless of race, gender, and education. However, the data cannot explain why SMI or poverty places these individuals at a greater risk or acknowledge diagnosis in relation to incarceration history.

Further study of incarceration in this underserved population will help explain and address the mechanisms by which mental illness places these individuals at greater risk of incarceration. Exploring the relationship among mental health diagnosis, treatment, crime, and poverty may help prevent future encounters with the CJS for these individuals.

## Abbreviations

CMHC: Community Mental Health Center
SMI: Serious Mental Illness
CJS: Criminal justice system
NLSY: National Longitudinal Survey of Youth
